# Allelic variations of HMW-GS and LMW-GS and quality analysis in Yannong series wheat cultivars/derivative lines

**DOI:** 10.3389/fgene.2024.1465540

**Published:** 2024-08-21

**Authors:** Nina Sun, Yanjun Mu, Dongmei Wang, Jiatong Li, Tangyu Yuan, Wei Liu, Ningning Yu, Xiaozhe Xu, Linzhi Li, Yuli Jin, Pengtao Ma

**Affiliations:** ^1^ Institute of Grain and Oil Crops, Yantai Academy of Agricultural Sciences, Yantai, China; ^2^ Yantai Key Laboratory of Characteristic Agricultural Biological Resources Conservation and Germplasm Innovative Utilization, College of Life Sciences, Yantai University, Yantai, China

**Keywords:** wheat, gluten quality, high-molecular-weight glutenin subunit, low-molecular-weight glutenin subunit, molecular markers

## Abstract

**Introduction:**

Gluten quality is one of the most important traits of the common wheat (*Triticum aestivum* L.). In Chinese wheat production, Yannong series cultivars/derivative lines possess unique characteristics and play an important role in both yield and quality contribution.

**Methods:**

To dissect their genetic basis of the gluten quality, in this study, allelic variations of high-molecular-weight glutenin subunit (HMW-GS) and low-molecular-weight glutenin subunit (LMW-GS) in 30 Yannong series wheat cultivars/derivative lines and three check cultivars were evaluated using the allele-specific molecular markers, and six crucial quality indexes were also further measured and analyzed.

**Results:**

The results demonstrated that the frequencies of HMW-GSs *By8*, *Dx5+Dy10* and *Dx5+Dy10+Dy12* in these 30 genotypes and three check cultivars accounted for 87.9%, 24.2% and 9.1%, respectively. For the allelic variations of LMW-GSs, *Glu-A3a*, *Glu-A3b*, *Glu-A3c*, *Glu-A3f*, and *Glu-A3g* were identified in 18, 9, 13, 11, and 2 genotypes, respectively; *Glu-B3d*, *Glu-B3g* and *Glu-B3f* were identified in 13, 23 and 4 genotypes, respectively. Notably, Yannong 999, containing *By8* + *Dx5* + *Dy10*, and Jinan 17 containing *By8* + *Dy12* both meet the national standard for high-quality wheat and belong to the category of first-class high-quality strong gluten wheat.

**Discussion:**

These findings can provide reference for wheat quality improvement and popularization in the production.

## Introduction

Wheat (*Triticum aestivum* L.) is an important food crop that provides about 20% of the calories for human consumption in the world (Wang et al., 2023; Han et al., 2024a). Protein is one of the organic compounds in wheat seeds, which accounts for 10%–12% of seed weight (Goesaert et al., 2005). The glutenin proteins play a key role in determining the quality of the wheat flour (Jin et al., 2015; Wang et al., 2018). They can be divided into four types based on the solubility: albumin dissolving in water and diluted buffer; globulin soluble dissolving in salt solution; gliadins dissolving in 70%–90% ethanol, and glutinin dissolving in dilute acid or alkali (Shwry et al., 1995). Under normal condition, albumin and globulin are considered as the metabolic proteins, accounting for about 15% of the glutenin proteins; whereas the remaining gliadins and glutinin are referred as the storage proteins, accounting for approximate 85%. It is reported that gliadin proteins mainly influence dough viscosity and extensibility, whereas the glutenin proteins were mainly involved in the process of dough cohesiveness and elasticity; and their relative proportions determine the distinct characteristics of the wheat gluten (Biesiekierski, 2017; Chen et al., 2018; Gao et al., 2021).

In wheat breeding and production, the quality trait is complex which is often influenced by multiple quality indexes, such as crude protein content, wet gluten content, water absorption, stability time, maximum resistance, stretch area, and bulk density (Ooms and Delcour, 2019). The content and composition of the glutenin proteins are important in determining various quality traits (Weegels et al., 1996). According to the molecular weight, wheat glutenin could be divided into high-molecular-weight glutenin subunits (HMW-GSs) and low-molecular-weight glutenin subunits (LMW-GSs), accounting for 7%–15% and 20%–35% of the storage protein, respectively (Peng et al., 2022). The diversified allelic variations of HMW-GS and LMW-GS play a crucial role in determining different processing quality of wheat flour, particularly affecting gluten strength and dough extensibility (Nagamine et al., 2000; Goesaert et al., 2005; Gao et al., 2016; Sherman et al., 2018; Jiang et al., 2019).

HMW-GSs are encoded by the *Glu-1* locus located on the long arms of homoeologous Group I chromosomes 1A, 1B and 1D, which were designated as *Glu-A1*, *Glu-B1* and *Glu-D1*, respectively (Payne et al., 1980). There are two closely linked genes at each locus: x-type and y-type subunits, and the molecular weight of x-type is higher than that of y-type (Shewry et al., 2003). Theoretically, there are six HMW-GSs in common wheat, however, 3-5 loci were usually expressed because of allelic variations and gene silencing (Yu et al., 2019). Different types of HMW-GSs have different impacts on the gluten quality (Jiang et al., 2019; Li et al., 2019). It was reported that variations in *Glu-D1* provided a greater contribution than that in *Glu-B1* or *Glu-A1* (Payne et al., 1980). The LMW-GSs are encoded by *Glu-3* locus that mapped on the short arms of homoeologous Group I chromosomes 1A, 1B and 1D, which were designated as *Glu-A3*, *Glu-B3* and *Glu-D3*, respectively (Jackson et al., 1996). The LMW-GSs in 222 wheat genotypes were analyzed using sodium dodecyl sulfate polyacrylamide gel electrophoresis (SDS-PAGE) and a total of 20 LMW-GS alleles were identified, including six alleles *a-f* at *Glu-A3* locus, nine alleles *a-i* at *Glu-B3* locus, and five alleles *a-e* at *Glu-D3* locus (Jackson et al., 1996). Due to the similar migration or overlap of LMW-GS alleles in SDS-PAGE detection, accurate identification of the LMW-GS is extremely difficult. With the development of molecular markers, the gene-specific markers were developed at *Glu-A3* and *Glu-B3* loci (Wang et al., 2009; 2010), but not at *Glu-D3* locus due to the rarely variations of the alleles (Zhao et al., 2006; Dai et al., 2020). Previous studies also showed that HMW-GS can explain 18%–55% of the variations in gluten strength and elasticity, while LMW-GS can explain 20% (Gianibelli et al., 2001; Li et al., 2019). For example, the HMW-GSs *1Ax1*, 1Ax2*, *1Bx17+1By18*, and *1Dx5+1Dy10* have been widely regarded as the high-quality subunits or subunit combinations, with positive effects on gluten strength and elasticity (Payne et al., 1987; Rasheed et al., 2019). The *Glu-A3b*, *Glu-B3b*, and *Glu-D3e* of LMW-GSs are the alleles that mainly contribute to wheat gluten strength (Gupta and acrichie, 1991).

Yannong series wheat cultivars, developed by Shandong Yantai Academy of Agricultural Sciences (Yantai, China), have unique characteristics in Chinese wheat production due to the unique ecological and climatic conditions in Yantai, China. Their promotion areas have reached 39.69 million hm^2^ in production (Liu et al., 2019). Lots of wheat cultivars have been developed using Yannong series cultivars as parents. For instance, the backbone parent Youbaomai is the first semi-dwarf high-yield cultivar with a yield exceeding 7,500 kg/hm^2^ in China; Yannong 15, as a high-yield and high-quality wheat cultivar, has been used in production more than 40 years; Yannong 999, a super-high yield and strong-gluten wheat cultivar, produced 12,255 kg/hm^2^ in the high-yield establishment of wheat and created the highest record of wheat yield in Shandong province and the highest winter wheat yield record in the national acceptance test (Xin et al., 2019).

To dissect the genetic basis of the gluten quality in Yannong series wheat cultivars/derivative lines, this study investigated the allelic variations of three key loci *Glu-1*, *Glu-A3*, and *Glu-B3* affecting gluten quality using molecular markers, measured their main quality related indexes, such as protein content, wet gluten content, development time and stability time, and also explored the relationship between the allelic variations and index of gluten quality. This study could provide insights in wheat quality improvement and popularization in the production.

## Materials and methods

### Plant materials and field trials

Thirty Yannong series wheat cultivars/derivative lines and three check cultivars were provided by Shandong Yantai Academy of Agricultural Sciences, Yantai, China. All these genotypes were sown at Yantai National Crop Variety Regional Test Station (37° 65′59″N, 120° 47′01″E) from 2022 to 2023 in a randomized complete block design with three replicates, and among them, wheat cultivars Chinese Spring, Jimai 22 and Jinan 17 which carried subunits *By8* and *Dy12* at *Glu-B1* and *Glu-D1* locus, respectively, were used as positive controls. Each cultivar/line was planted as a plot with three rows (1.5 m length and 0.25 m between rows) and 30 seeds per row. The field management was the same as the local field production at Yantai National Crop Variety Regional Test Station. After normal maturity, each wheat genotype was separately harvested. After the grains moisture were less than 13%, phenotypes of the quality traits were gained with three replicates for each genotype.

### Quality index determination

The wet gluten content, protein content, and stability time, water absorption, development time, and sedimentation value traits were set as the key factors which significantly influenced wheat quality. These six crucial quality indexes of the 30 Yannong series wheat cultivars/lines and three check cultivars were determined using the near-infrared instrument Infratec TM 1241 (Foss, Denmark) to scan NIR spectra. WinISI II v1.50 (InfraSoft International LLC, 2000) software was used to find out the final reading (Irshad et al., 2023). All the experiments were repeated three times.

### Genotying of gluten quality related genes using gene-specific molecular markers

Genomic DNA of the 33 wheat genotypes were extracted from their young leaf using the modified cetyltrimethylammonium bromide (CTAB) method (Sharp et al., 1988). Then, they were tested by using 20 diagnostic markers for 21 gluten quality trait genes or gene combinations (HMW-GSs subunit alleles *By8*, *Bx14*, *Dx5*, *Dy10* and *Dy12*; LMW-GSs subunit alleles *Glu-A3a*, *Glu-A3b*, *Glu-A3ac*, *Glu-A3d*, *Glu-A3e*, *Glu-A3f*, *Glu-A3g*, *Glu-B3a*, *Glu-B3b*, *Glu-B3c*, *Glu-B3d*, *Glu-B3e*, *Glu-B3f*, *Glu-B3g*, *Glu-B3h*, and *Glu-B3i*) (Table 1).

**TABLE 1:** Molecular markers for the detection of high and low molecular weight glutenin subunits.

Genes	Markers	Primer sequence (5’→3’)	Target bands (bp)	References
*By8*	ZSBy8F5/ZSBy8R5	F: TTAGCGCTAAGTGCCGTCT R: TTGTCCTATTTGCTGCCCTT	527	Lei et al. (2006)
*Bx14*	AQBx14-FIAQBx14-R	F: GCAGCAACTCCAACAAATG R: CTTGGCCTGGATAGTATGAC	407	Xu et al. (2006)
*Dx5*	Dx5-F/Dx5-R	F: CGTCCCTATAAAAGCCTAGC R: AGTATGAAACCTGCTGCGGAC	478	Liu et al. (2008)
*Dy10*	UMN26-F/UMN26-R	F: CGCAAGACAATATGAGCAAACT R: TTGCCTTTGTCCTGTGTGC	397	Liu et al. (2008)
*Dyl2*	UMN26-F/UMN26-R	F: CGCAAGACAATATGAGCAAACT R: TTGCCTTTGTCCTGTGTGC	415	Liu et al. (2008)
*Glu-A3a*	LAlF/SAlR	F: AAA​CAG​AAT​TAT​TAA​AGC​CGG R: GGTTGTTGTTGTTGCAGCA	529	Wang et al. (2009)
*Glu-A3b*	LA3F/SA2R	F: TTC​AGA​TGC​AGC​CAA​ACA​A R: GCTGTGCTTGGATGATACTCTA	894	Wang et al. (2009)
*Glu-A3ac*	LAlF/SA3R	F: AAA​CAG​AAT​TAT​TAA​AGC​CGG	573	Wang et al. (2009)
		R: GTG​GCT​GTT​GTG​AAA​ACG​A		
*Glu-A3d*	LA3F/SA4R	F: TTCAGATGCAGCCAAACAA	967	Wang et al. (2009)
		R: TGG​GGT​TGG​GAG​ACA​CAT​A		
*Glu-A3e*	LAlF/SA5R	F: AAA​CAG​AAT​TAT​TAA​AGC​CGG R: GGCACAGACGAGGAAGGTT	158	Wang et al. (2009)
*Glu-A3f*	LAlF/SA6R	F: AAACAGAATTATTAAAGCCGG	552	Wang et al. (2009)
		R: GCT​GCT​GCT​GCT​GTG​TAA​A		
*Glu-A3g*	LAlF/SA7R	F: AAACAGAATTATTAAAGCCGG	1,345	Wang et al. (2009)
		R: AAA​CAA​CGG​TGA​TCC​AAC​TAA		
*Glu-B3a*	SB1F/SB1R	F: CAC​AAG​CAT​CAA​AAC​CAA​GA	1,095	Wang et al. (2010)
		R: TGG​CAC​ACT​AGT​GGT​GGT​C		
*Glu-B3b*	SB2F/SB2R	F: ATC​AGG​TGT​AAA​AGT​GAT​AG	1,570	Wang et al. (2010)
		R: TGC​TAC​ATC​GAC​ATA​TCC​A		
*Glu-B3c*	SB3F/SB3R	F: CAAATGTTGCAGCAGAGA	472	Wang et al. (2010)
		R: CAT​ATC​CAT​CGA​CTA​AAC​AAA		
*Glu-B3d*	SB4F/SB4R	F: CAC​CAT​GAA​GAC​CTT​CCT​CA	662	Wang et al. (2010)
		R: GTT​GTT​GCA​GTA​GAA​CTG​GA		
*Glu-B3e*	SB5F/SB5R	F: GAC​CTT​CCT​CAT​CTT​CGC​A	669	Wang et al. (2010)
		R: GCAAGACTTTGTGGCATT		
*Glu-B3fg*	SB6F/SB6R	F: TAT​AGC​TAG​TGC​AAC​CTA​CCA​T R: CAACTACTCTGCCACAACG	812	Wang et al. (2010)
*Glu-B3g*	SB7F/SB7R	F: CCAAGAAATACTAGTTAACACTAGTC R: GTTGGGGTTGGGAAACA	853	Wang et al. (2010)
*Glu-B3h*	SB8F/SB8R	F: CCA​CCA​CAA​CAA​ACA​TTA​A	1,022	Wang et al. (2010)
		R: GTG​GTG​GTT​CTA​TAC​AAC​GA		
*Glu-B3i*	SB9F/SB9R	F: TATAGCTAGTGCAACCTACCAT	621	Wang et al. (2010)
		R: TGG​TTG​TTG​CGG​TAT​AAT​TT		

The PCR amplification system was referred to the reported ones (Han et al., 2023; 2024b) with moderate modifications: a 10 μL volume was used for PCR amplification, including 1 μL 50 ng/μL template DNA, 5 μL 2 × Taq Master Mix (Vazyme P112-03, China) and 0.5 μL 10 μM/μL primers, adding ddH_2_O to 10 μL. The PCR amplification procedure was as follows: pre-denaturation at 94°C for 5 min, denaturation at 94°C for 30 s, annealing at 50°C–65°C for 1 min (depending on different primers), extension at 72°C for 40–120 s (depending on different target bands); 30-36 cycles were performed in total; PCR amplification was prolonged for 10 min at 72°C and stored at 4°C. The PCR products were separated in either 8% non-denaturing polyacrylamide gels with 19:1, 29:1 or 39:1 ratios of acrylamide and bis-acrylamide, then silver stained and visualized as previously described (Santos et al., 1993; Jin et al., 2024), or 1.5% agarose gel, then visualized using the Gel Documentation System (Gel Doc XR+, BIO-RAD, Hercules, CA, United States) (Gebrewahid et al., 2020).

### Descriptive statistics and correlation analysis

The phenotype and genotype data were analyzed using the SPSS 19.0 software (IBM, Chicago, United States). Descriptive statistics were employed to assess the variability of the examined parameters (i.e., the means, maximum, minimum, and standard deviations). Coefficients of variation (CV) also were calculated as part of the analysis of variation.

## Results

### Allelic variations of HMW-GS

**FIGURE 1:**
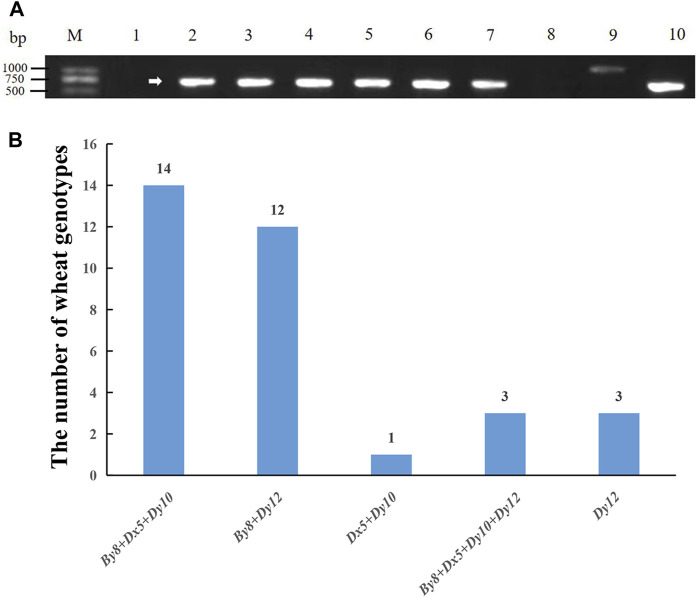
PCR amplification of molecular marker *ZSBy8F5/ZSBy8R5* **(A)**, and distribution of high molecular weight glutenin subunits **(B)** in 30 Yannong wheat cultivars/derivative lines and three check cultivars Chinese Spring, Jinan 17 and Jimai 22. M: DL2000, 1–10: Yannong 15, Yannong 999, Yannong 215, Yannong 377, Yannong 836, Yannong 5158, Yannong 1212, Yannong 999/LS4223, Yannong 999/DH5133, Hang 2. The white arrow indicates the specific amplified band.

The types of HMW-GS loci in the 30 Yannong series wheat genotypes were identified and analyzed using diagnostic markers using Chinese Spring, Jimai 22 and Jinan 17 as positive checks. Among the 33 tested wheat genotypes, the marker *ZSBy8F5/ZSBy8R5* amplified the 527 bp band in 26 genotypes except for Yannong 15, Yannong 999/LS4223, Yannong 999/DH5133 and Yannong 5158/Yannong 15, suggesting the predominant subunit *By8* at *Glu-B1* locus (86.7%). At the *Glu-D1* locus, *Dx5-F/Dx5-R* amplified 281 bp band in 18 genotypes, *UMN26-F/UMN26-R* amplified 397 bp and 415 bp bands in 18 and 15 genotypes, respectively. Therefore, 60.0%, 50.0% and 60.0% of these genotypes carry the subunits *Dx5*, *Dy10* and *Dy12*, respectively (Figure 1; Table 2). The *Dx5+Dy10* alleles on *Glu-D1* locus was an elite allele composition for improving bread-making quality. In addition, none of genotypes carry the subunit *Bx14* at the *Glu-B1* locus.

**TABLE 2:** Distribution of high and low molecular weight glutenin subunits in 30 Yannong wheat cultivars/derivative lines and three check cultivars Chinese Spring, Jinan 17 and Jimai 22.

Cultivars/derivative lines	Subunits
Yannong 999	By8, Dx5, Dy10, Glu-A3a, Glu-B3f
Yannong 215	By8, Dyl2, Glu-A3a, Glu-B3d, Glu-B3f
Yannong 199	By8, Dx5, Dy10, Glu-A3d, Glu-B3f
Yannong 377	By8, Dyl2, Glu-A3a, Glu-B3g
Yannong 836	By8, Dyl2, Glu-A3c, Glu-B3d
Yannong 5158	By8, Dx5, Dy10, Glu-A3c, Glu-B3d
YanBlu14-15	By8, Dyl2, Glu-A3a, Glu-B3g
Yannong 672	By8, Dx5, Dy10, Glu-A3c, Glu-A3d
Yannong 9292	By8, Dx5, Dy10, Glu-A3d
Hang 2	By8, Dyl2, Glu-A3a, Glu-B3d
Yannong 1212	By8, Dy12, Glu-A3b, Glu-A3c, Glu-A3d, Glu-B3d, Glu-B3g
Yannong 15	Dyl2, Glu-A3b, Glu-A3c, Glu-B3g
Yannong 999/Huaimai 33	By8, Dx5, Dy10, Dyl2, Glu-A3a, Glu-A3b, Glu-A3d, Glu-A3f, Glu-B3g
Yannong 999/Jinai 176	By8, Dx5, Dy10, Glu-A3a, Glu-B3g
Yannong 999/Kongmai 181	By8, Dx5, Dy10, Dyl2, Glu-A3a, Glu-B3g
Yannong 999/LS4223	Dx5, Dy10, Glu-A3c, Glu-A3d, Glu-A3f, Glu-A3g, Glu-B3g
Yannong 999/Jimai 22	By8, Dyl2, Glu-A3a, Glu-A3b, Glu-A3d, Glu-A3f, Glu-B3d, Glu-B3g
Yannong 999/Zhoumai 28	By8, Dx5, Dy10, Glu-A3a, Glu-A3b, Glu-A3d, Glu-A3f, Glu-A3g, Glu-B3d
Yannong 999/DH5133	Dyl2, Glu-A3a, Glu-A3b, Glu-A3d, Glu-B3d, Glu-B3g
Zhengmai 366/Yannong 999	By8, Dx5, Dy10, Dyl2, Glu-A3a, Glu-A3b, Glu-A3d, Glu-B3d, Glu-B3g
Taishan 4241/Yannong 999	By8, Dx5, Dy10, Glu-A3a, Glu-A3d, Glu-A3f, Glu-B3g
Yannong 999/BY18	By8, Dx5, Dy10, Glu-A3a, Glu-B3d, Glu-B3g
Yannong 999/Jinong 19	By8, Dx5, Dy10, Glu-A3a, Glu-A3f, Glu-B3g
Yannong 999/Zhoumai 27	By8, Dyl2, Glu-A3a, Glu-A3f, Glu-B3g
Zhoumai 27/Yannong 999	By8, Dyl2, Glu-A3c, Glu-A3d, Glu-A3f, Glu-B3g
Qianhemai 17/Yannong 999	By8, Dx5, Dy10, Glu-A3a, Glu-A3f, Glu-B3g
Yannong 999/Xu 748	By8, Dx5, Dy10, Glu-A3c, Glu-A3d, Glu-A3f, Glu-B3g
Yannong 836/Jimai 22	By8, Dx5, Dy10, Glu-A3c, Glu-A3f, Glu-B3d, Glu-B3g
Yannong 5158/Jimai 22	By8, Dx5, Dy10, Glu-A3b, Glu-A3c, Glu-A3d, Glu-B3d, Glu-B3g
Yannong 5158/Yannong 15	Dyl2, Glu-A3c, Glu-B3d, Glu-B3g
Chinese Spring	By8, Dyl2, Glu-A3a, Glu-A3d, Glu-B3g
Jinan 17	By8, Dyl2, Glu-A3c, Glu-A3d, Glu-B3f
Jimai 22	By8, Dyl2, Glu-A3b, Glu-A3c, Glu-A3d, Glu-B3g

### Allelic variations of LMW-GS

**FIGURE 2:**
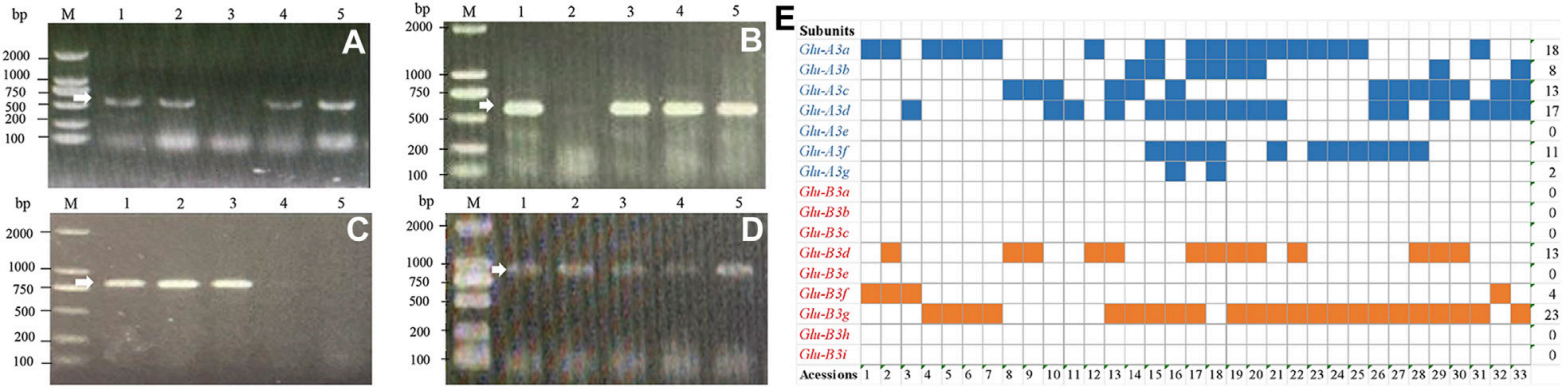
PCR amplification of molecular markers *LA1F/SA1R* **(A)**, *LA1F/SA3R* **(B)**, *SB6F/SB6R* **(C)**, and *SB7F/SB7R* **(D)** and distribution of low molecular weight glutenin subunits **(E)** in 30 Yannong wheat cultivars/derivative lines and three check cultivars Chinese Spring, Jinan 17 and Jimai 22. M: DL2000, 1–5 **(A)**: Yannong 999, Yannong 215, Yannong 377, Yannong 836, Yannong 5158; 1–5 **(B–C)**: Yannong 999, Yannong 199, Yannong 377, Yannong 836, Yannong 5158; **(D)** 1–5: Yannong 377, Yannong 1212, Yannong 15, Yannong 999/Huaimai 33, Yannong 999/Kongmai 181. The white arrow indicates the specific amplified fragment. **(E)** Acessions 1–33: Yannong 999, Yannong 215, Yannong 199, Yannong 377, Yannong 836, Yannong 5158, YanBlu14-15, Yannong 672, Yannong 9292, Hang 2, Yannong 1212, Yannong 15, Yannong 999/Huaimai 33, Yannong 999/Jinai 176, Yannong 999/Kongmai 181, Yannong 999/LS4223, Yannong 999/Jimai 22, Yannong 999/Zhoumai 28, Yannong 999/DH5133, Zhengmai 366/Yannong 999, Taishan 4241/Yannong 999, Yannong 999/BY18, Yannong 999/Jinong 19, Yannong 999/Zhoumai 27, Zhoumai 27/Yannong 999, Qianhemai 17/Yannong 999, Yannong 999/Xu 748, Yannong 836/Jimai 22, Yannong 5158/Jimai 22, Yannong 5158/Yannong 15, Chinese Spring, Jinan 17, Jimai 22. Blue squares indicate presence of gene *Glu-A3* and orange squares indicate presence of gene *Glu-B3*. Blank squares indicate absence of the tested genes.

At the *Glu-A3* locus, seven markers were used to detect 30 Yannong series wheat genotypes using Chinese Spring, Jimai 22 and Jinan 17 as positive checks. The results showed that the markers *LA1F/SA1R* for *Glu-A3a*, *LA3F/SA2R* for *Glu-A3b*, *LA1F/SA3R* for *Glu-A3c*, *LA1F/SA6R* for *Glu-A3f*, and *LA1F/SA7R* for the *Glu-A3g* locus amplified the target bands in 18, 9, 13, 11, and 2 genotypes, respectively, indicating that they carried the responding alleles at the *Glu-A3* locus (Figures 2A, B, E; Table 2). Additionally, no target bands were detected using the marker *LA1F/SA5R* for *Glu-A3e* in all the tested genotypes.

At the *Glu-B3* locus, nine markers were also used to test 30 Yannong series wheat genotypes using Chinese Spring, Jimai 22 and Jinan 17 as positive checks. Among them, the marker *SB4F/SB4R* for *Glu-B3d* amplified 662 bp band in 13 genotypes. The marker *SB7F/SB7R* for *Glu-B3g* amplified 853 bp band in 23 genotypes and *SB6F/SB6R* for *Glu-B3fg* amplified 812 bp band in 27 genotypes. It was concluded that four wheat cultivars Yannong 999, Yannong 215, Yannong 199 and Jinan 17 carried *Glu-B3f* allele. The markers *SB1F/SB1R* for *Glu-B3a*, *SB2F/SB2R* for *Glu-B3b*, *SB3F/SB3R* for *Glu-B3c*, *SB5F/SB5R* for *Glu-B3e*, *SB8F/SB8R* for *Glu-B3h* did not amplify the target bands in all the tested wheat genotypes, suggesting that these alleles were absent in these genotypes (Figures 2C–E; Table 2).

### Index of the gluten quality traits

**FIGURE 3:**
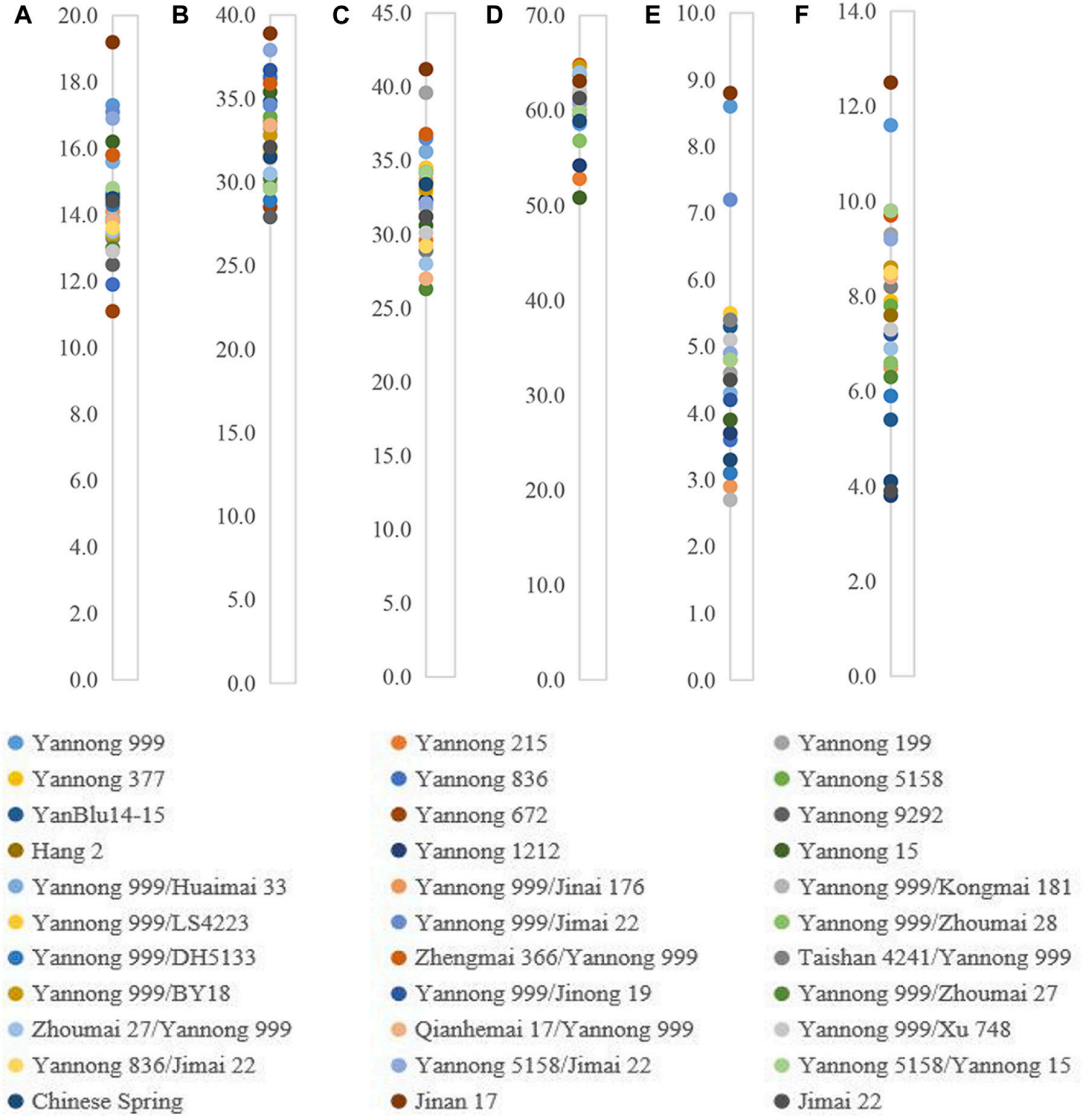
Distribution of six crucial quality indexes protein content (%) **(A)**, wet gluten content (%) **(B)**, sedimentation value (mL) **(C)**, water absorption (mL/g) **(D)**, development time (min) **(E)**, and stability time (min) **(F)** in 30 Yannong series wheat cultivars/derivative lines and three check cultivars Chinese Spring, Jinan 17 and Jimai 22.

For these 30 Yannong series wheat genotypes and three check cultivars, the protein content ranged from 11.1% to 19.2%; wet gluten content from 27.9% to 38.9%; development time from 2.7 to 8.8 min, and stability time from 3.8 to 12.5 min, respectively. Water absorption and sedimentation value ranged from 50.8 to 64.8 mL/g and 26.3–41.2 mg, respectively. The variance analysis revealed that these indexes were significantly different among 30 Yannong series wheat genotypes and three check cultivars (Figure 3; Table 3). Notably, Yannong 999 with the protein content 17.3%, wet gluten content 36.2%, water absorption 58.6 mL/g, development time 8.6 min, and stability time 11.6 min, and Jinan 17 with these indexes of 19.2%, 38.9%, 63.1 mL/g, 8.8 min and 12.5 min, both meet the national standards for high-quality wheat and belong to the category of first-class high-quality strong gluten wheat.

**TABLE 3:** Quality indexes of 30 Yannong wheat cultivars/derivative lines and three check cultivars Chinese Spring, Jinan 17 and Jimai 22.

Cultivars/lines	Protein content (%)	Wet gluten content (%)	Sedimentation Value (mL)	Water absorption (mL/g)	Development time (min)	Stability time (min)
Yannong 999	17.3	36.2	36.6	58.6	8.6	11.6
Yannong 215	14.1	33.6	29.6	52.8	3.7	7.6
Yannong 199	14.6	33.2	39.6	63.8	4.6	9.3
Yannong 377	13.8	31.5	32.8	61.2	3.1	7.9
Yannong 836	11.9	36.3	28.9	59.7	3.6	4.1
Yannong 5158	15.6	33.9	35.6	60.2	5.3	7.8
YanBlu14-15	15.8	34.8	33.5	59.6	5.3	5.4
Yannong 672	11.1	28.5	31.2	60.5	4.8	6.5
Yannong 9292	12.5	27.9	31.2	60.2	3.9	6.5
Hang 2	13.9	31.5	34.5	60.2	3.1	7.6
Yannong 1212	13.8	29.7	32.3	54.2	3.7	3.8
Yannong 15	16.2	35.4	30.6	50.8	3.9	8.6
Yannong 999/Huaimai 33	15.6	34.6	35.6	61.2	4.3	5.9
Yannong 999/Jinan 1076	14.1	29.8	33.7	60.8	2.9	6.5
Yannong 999/Kongmai 181	13.9	29.8	31.8	62.8	2.7	6.9
Yannong 999/LS4223	14.5	31.8	34.5	61.7	5.5	6.9
Yannong 999/Jimai 22	17.1	34.6	36.5	60.2	7.2	9.8
Yannong 999/Zhoumai 28	14.7	32.8	29.2	56.8	5.4	6.6
Yannong 999/DH51303	14.3	28.9	34.2	61.8	3.1	5.9
Zhengmai 366/Yannong 999	15.8	35.9	36.8	64.8	4.5	9.7
Taishan 4241/Yannong 999	13.3	29.6	28.9	59.7	5.4	8.2
Yannong 999/BY18	13.4	32.8	33.1	64.6	3.3	8.6
Yannong 999/Jimai 19	14.6	36.7	34.2	60.5	4.2	7.2
Yannong 999/Zhoumai 27	13	30.2	26.3	63.8	4.8	6.3
Zhoumai 27/Yannong 999	13.5	30.5	28	64	4.9	6.9
Qianhemai 17/Yannong 999	13.8	33.4	27	61.6	4.8	8.4
Yannong 999/Xu 7048	12.9	31.4	30.1	62.1	5.1	7.3
Yannong 836/Jimai 22	13.6	32.1	29.2	60.4	4.8	8.5
Yannong 5158/Jimai 22	16.9	37.9	32.1	60.8	4.9	9.2
Yannong 5158/Yannong 15	14.8	29.6	34.2	59.8	4.8	9.8
Chinese Spring	14.5	31.5	33.4	58.9	3.3	4.1
Jinan 17	19.2	38.9	41.2	63.1	8.8	12.5
Jimai 22	14.4	32.1	31.2	61.3	4.5	3.9

### Relationship between the genotypes and phenotypes

The *Dx5+Dy10* composition has been identified as an elite alleles composition for improving bread-making quality. In the present study, 18 of 30 Yannong series wheat cultivars or derivative lines carried *Dx5+Dy10* composition, indicating it will be necessary to strengthen positive selection for the *Dx5+Dy10* composition for the genetic improvement of high-quality wheat cultivars. Wheat cultivars Yannong 999, Jinan 17, Yannong 377 and Yannong 199 were identified to possess *Glu-B3f* locus for LMW-GS, and their protein and wet gluten contents were more than 15% and 35% (the first-class high-quality gluten standard), respectively, suggesting their elite gluten characteristics (Figure 3; Table 3). Additionally, the stability time of these genotypes containing *Dx5* locus for HMW-GS and *Glu-B3g* locus for LMW-GS were longer than the others, such as Yannong 999 (11.6 min), Yannong 199 (9.3 min), and Yannong 5158 (7.8 min). So, they meet the second-class high-quality gluten standard of 7.0 min.

## Discussion

For a long time, wheat breeding mainly focused on improving the yield, and hence neglected the pursuit of developing high quality cultivars, leading to the disconnect between wheat supply and demand and the lacking coexistence of high yield and high quality. Therefore, strengthening the breeding and production ability of high-quality wheat cultivars is of great significance for improving food security, solving food conflicts, and enhancing people’s sense of happiness. In China, Yannong series wheat cultivars have unique characteristics, such as super-high yield and the harmonious improvement between the yield and quality traits. For instance, Yannong 999, one of the representatives of the Yannong series wheat cultivars, concurrently possesses elite super-high yield and high quality performance, which reached the Chinese strong gluten wheat standard for two consecutive years in the regional test of the national Huang Huai southern winter water group. To explore genetic basis of the high quality in Yannong series wheat cultivars and their derivative lines, molecular markers for the related genes to quality were used to detect their HMW-GSs and LMW-GSs, and we also combined with the main quality phenotype indexes to dissect the grain quality traits. Determining the alleles composition conferring the quality traits could contribute to both wheat quality breeding and popularization using Yannong series wheat cultivars/derivative lines.

In wheat grain quality traits, although HMW-GS accounts for only 10% of the storage protein content, it is a key factor affecting wheat processing quality (Payne et al., 1987; Zhang et al., 2009). Previous studies proved that the *Glu-D1* locus has the greatest effect on the processing quality of the wheat flour, while *Glu-B1* and *Glu-A1* have relatively less effect. The different protein subunit compositions showed diversified effects on the processing quality of wheat flour. Among them, 1, 2*, 5 + 10 are high-quality protein subunits or subunit compositions for improving the quality of gluten (Liu et al., 2005; Lu et al., 2017); 2 + 12 subunit composition associated with poor quality were the most common subunit composition in 123 regional and modern bread wheat cultivars, accounting for 63.4%, and 5 + 10 subunit composition associated with strong gluten bread wheat were found in 22 wheat cultivars, accounting for only 17.8%. Besides, 2.1 + 12, 2 + 12′, and 2 + 12* subunit compositions were also found among these wheat cultivars (Sönmez et al., 2023). Meanwhile, a previous study showed that *Glu-B1* had the highest effect on the variations for the gluten, dough and end-use quality traits, whereas *Glu-A1* and *Glu-D3* had the lowest impact. The *Glu-D1* locus had a strong impact on gluten strength but its contribution to either SDS-Sedimentation volume, gluten extensibility and bread loaf volume was minimal (Guzmán et al., 2022). Jin et al. (2015) found that HMW-GS has a positive effect on dough strength, percentage of insoluble gluten aggregates, and total score of bread. Wheat genotypes containing subunits *By9*, *Bx17*, and *Dx5* have the highest dough strength and percentage of insoluble gluten aggregates; the genotypes carrying *Ax1*, *Ax2*, and *Dx5* have the highest total score for the bread. In this present study, the frequencies of subunit compositions *Dx5+Dy10* and *Dx5+Dy10+Dy12* in 30 Yannong series wheat genotypes and three check cultivars accounted for 24.2% and 9.1%, respectively. Further analysis revealed significant greater ratings for stability time among wheat cultivars carrying *Dx5+Dy10*, which was consistent with the report by Jin et al. (2015). Despite an increasing trend in the selection frequency of *Dx5+Dy10* in recent years, there is still potential for further improvement. Notably, Yannong 999 containing subunit composition *By8*+*Dx5+Dy10* and Jinan 17 with subunit composition *By8*+*Dy12* both meet the national standard for high-quality wheat and belong to the category of first-class high-quality strong gluten wheat. Therefore, they can be popularized in large area for high quality wheat production, and also used as elite breeding parents for high quality wheat breeding.

In analyzing the constitution of the protein subunits, there are certain difficulties in using traditional SDS-PAGE to analyze LMW-GS in hexaploid common wheat: firstly, there are more allelic variations in LMW-GS; secondly, LMW-GS exhibits similar electrophoretic mobility or overlap with alcohol content proteins (Jackson et al., 1996). With the development of molecular markers, a set of STS markers, including seven alleles (*Glu-A3a-g*) and nine alleles (*Glu-B3a-i*) have been successively developed (Wang et al., 2009; Wang et al., 2010). Meanwhile, six alleles at *Glu-D3* locus have not been developed the detected markers due to their small variations (Zhao et al., 2006; Dai et al., 2020). Using these markers, LMW-GS variations in 343 wheat cultivars from Xinjiang, China were analyzed, and two new types at the *Glu-A3* locus, named *Glu-A3new1* and *Glu-A3new2*, two new types at the *Glu-B3* locus, named *Glu-B3new3* and *Glu-B3new4*, were identified (Dai et al., 2020). In the present study, we revealed the allelic variations of LMW-GS in 30 Yannong series wheat genotypes and three check cultivars. At the *Glu-A3* locus, *Glu-A3a*, *Glu-A3b*, *Glu-A3c*, *Glu-A3f*, and *Glu-A3g* were identified in 18, 9, 13, 11 and two wheat cultivars/lines, respectively. At the *Glu-B3* locus, *Glu-B3d*, *Glu-B3g* and *Glu-B3f* were identified in 13, 23 and four genotypes, respectively. *Glu-A3d*, *Glu-A3e*, *Glu-B3a*, *Glu-B3b*, *Glu-B3c*, *Glu-B3e*, and *Glu-B3h* were absent in all the tested 33 genotypes. These information are valuable for the popularization of these genotypes and breeding improvement using these genotypes as parents.

In the present study, allelic variations of *Glu-1*, *Glu-A3*, *Glu-B3*, and six crucial quality indexes were identified in 30 Yannong series wheat cultivars/derivative lines and three check cultivars. However, the quality traits are complex which are often influenced by multiple quality indexes (Ooms and Delcour, 2019). In future, more quality related genes should be analyzed to identify the genetic basis of the high quality in Yannong series wheat cultivars/derivative lines, such as wheat yellow pigment, polyphenol oxidase activity, and grain (LOX) activity. Additionally, during the process of wheat high-quality breeding, more attention could be paid to combine different high quality subunits, evaluate the impact of different combinations on wheat quality and other breeding traits, and finally, find the optimal combinations for high-quality breeding.

## Conclusion

In conclusion, the present study determined the allelic variations of *Glu-1*, *Glu-A3*, and *Glu-B3*, measured the quality related traits, and explored the relationship between allelic combinations and quality performance in 30 Yannong series wheat cultivars/derivative lines and three check cultivars. Therefore, this study could provide reference information for modern wheat quality improvement and popularization in the production.

## Data Availability

The original contributions presented in the study are included in the article/supplementary material, further inquiries can be directed to the corresponding authors.

## References

[B1] Biesiekierski J. (2017). What is gluten? J. Gastroenterol. Hepatol. 32, 78–81. 10.1111/jgh.13703 28244676

[B2] Chen G. Ehmke L. Miller R. Faa P. Smith G. Li Y. (2018). Effect of sodium chloride and sodium bicarbonate on the physicochemical properties of soft wheat flour doughs and gluten polymerization. J. Agric. Food Chem. 66, 6840–6850. 10.1021/acs.jafc.8b01197 29879838

[B3] Dai Y. Xu D. Yan Y. Wen Z. Zhang J. Chen H. (2020). Characterization of high- and low-molecular-weight glutenin subunits from Chinese Xinjiang wheat landraces and historical varieties. J. Food Sci. Technol. 57, 3823–3835. 10.1007/s13197-020-04414-5 32904055 PMC7447723

[B4] Gao X. Liu T. Yu J. Li L. Feng Y. Li X. (2016). Influence of high-molecular-weight glutenin subunit composition at *Glu-B1* locus on secondary and micro structures of gluten in wheat (*Triticum aestivum* L.). Food Chem. 197, 1184–1190. 10.1016/j.foodchem.2015.11.085 26675856

[B5] Gao Y. An K. X. Guo W. W. Chen Y. M. Zhang R. J. Zhang X. (2021). The endosperm-specific transcription factor *TaNAC019* regulates glutenin and starch accumulation and its elite allele improves wheat grain quality. Plant Cell 33, 603–622. 10.1093/plcell/koaa040 33955492 PMC8136912

[B6] Gebrewahid T. Zhang P. Yao Z. Li Z. Liu D. (2020). Identification of leaf rust resistance genes in bread wheat cultivars from Ethiopia. Plant Dis. 104, 2354–2361. 10.1094/pdis-12-19-2606-re 32697658

[B7] Gianibelli M. Larroque O. Macritchie F. Wrigley C. (2001). Biochemical, genetic, and molecular characterization of wheat glutenin and its component subunits. Cereal Chem. 78, 635–646. 10.1094/cchem.2001.78.6.635

[B8] Goesaert H. Brijs K. Veraverbeke W. Courtin C. Gebruers K. Delcour J. (2005). Wheat flour constituents: how they impact bread quality, and how to impact their functionality. Trends Food Sci. Technol. 16, 12–30. 10.1016/J.TIFS.2004.02.011

[B9] Gupta R. MacRitchie F. (1991). A rapid one-step one-dimensional SDS-PAGE procedure for analysis of subunit composition of glutenin in wheat. J. Cereal Sci. 14, 105–109. 10.1016/S0733-5210(09)80130-6

[B10] Guzmán C. Crossa J. Mondal S. Govindan V. Huerta J. Crespo-Herrera L. (2022). Effects of glutenins (*Glu-1* and *Glu-3*) allelic variation on dough properties and bread-making quality of CIMMYT bread wheat breeding lines. Field Crops Res. 284, 108585. 10.1016/J.FCR.2022.108585

[B11] Han G. Liu H. Zhu S. Gu T. Cao L. Yan H. (2024a). Two functional CC-NBS-LRR proteins from rye chromosome 6RS confer differential age-related powdery mildew resistance to wheat. Plant Biotechnol. J. 22, 66–81. 10.1111/pbi.14165 38153293 PMC10754004

[B12] Han G. Wang J. Yan H. Cao L. Liu S. Li X. (2023). Development and molecular cytogenetic identification of a new wheat-rye 6RL ditelosomic addition and 1R (1B) substitution line with powdery mildew resistance. J. Integr. Agric. 10.1016/j.jia.2023.10.004

[B13] Han G. Wang J. Yan H. Gu T. Cao L. Liu S. (2024b). Development and identification of two novel wheat-rye 6R derivative lines with adult-plant resistance to powdery mildew and high-yielding potential. Crop J. 12, 308–313. 10.1016/j.cj.2023.09.003

[B14] Irshad A. Guo H. Xiong H. Xie Y. Jin H. Gu J. (2023). Evaluation of altered starch mutants and identification of candidate genes responsible for starch variation in wheat. BMC Plant Biol. 23, 377. 10.1186/s12870-023-04389-3 37528349 PMC10391901

[B15] Jackson E. Morel M. Sontag-Strohm T. Branlard G. Metakovsky E. Redaelli R. (1996). Proposal for combining the classification systems of alleles of *Gli-1* and *Glu-3* loci in bread wheat (*Triticum aestivum* L.). J. Genet. Breed. 50, 321–336.

[B16] Jiang P. Xue L. Duan Y. Gu Y. Mu J. Han S. (2019). Effects of high-molecular-weight glutenin subunit combination in common wheat on the quality of crumb structure. J. Sci. Food Agric. 99, 1501–1508. 10.1002/jsfa.9323 30129098

[B17] Jin H. Wang Z. Li D. Wu P. Dong Z. Rong C. (2015). Genetic analysis of chromosomal loci affecting the content of insoluble glutenin in common wheat. J. Genet. Genomics 42, 495–505. 10.1016/j.jgg.2015.04.010 26408094

[B18] Jin Y. Han G. Zhang W. Bu B. Zhao Y. Wang J. (2024). Evaluation and genetic dissection of the powdery mildew resistance in 558 wheat accessions. New Crops 1, 100018. 10.1016/j.ncrops.2024.100018

[B19] Lei Z. Gale K. He Z. Gianibelli C. Larroque O. C. Xia X. (2006). Y-type gene specific markers for enhanced discrimination of high-molecular weight glutenin alleles at the *Glu-B1* locus in hexaploid wheat. J. Cereal Sci. 43, 94–101. 10.1016/j.jcs.2005.08.003

[B20] Li S. Liu Y. Tong J. Yu L. Ding M. Zhang Z. (2019). The overexpression of high-molecular-weight glutenin subunit *Bx7* improves the dough rheological properties by altering secondary and micro-structures of wheat gluten. Food Res. Int. 130, 108914. 10.1016/j.foodres.2019.108914 32156364

[B21] Liu J. Yu J. Wang P. Sun L. Sun N. Feng Y. (2019). Research progress and prospect of Yannong series wheat. China Seed Ind. 11, 18–22. 10.19462/j.cnki.1671-895x.20191023.021

[B22] Liu L. He Z. Yan J. Zhang Y. Xia X. Pena R. (2005). Allelic variation at the *Glu-1* and *Glu-3* loci, presence of the 1B.1R translocation, and their effects on mixographic properties in Chinese bread wheats. Euphytica 142, 197–204. 10.1007/s10681-005-1682-4

[B23] Liu S. Chao S. Anderson J. (2008). New DNA markers for high molecular weight glutenin subunits in wheat. Theor. Appl. Genet. 118, 177–183. 10.1007/s00122-008-0886-0 18797838

[B24] Lu J. Pang L. Chai S. (2017). Effects of HMW-GS on quality properties of spring wheat and e-valuation of subunit score system. J. Nucl. Agric. Sci. 31, 80–87. 10.11869/j.issn.100-8551.2017.01.0080

[B25] Nagamine T. Kai Y. Takayama T. Yanagisawa T. Taya S. (2000). Allelic variation at the *Glu-1* and *Glu-3* loci in southern Japanese wheats, and its effects on gluten properties. J. Cereal Sci. 32, 129–135. 10.1006/jcrs.2000.0323

[B26] Ooms N. Delcour J. (2019). How to impact gluten protein network formation during wheat flour dough making. Curr. Opin. Food Sci. 25, 88–97. 10.1016/j.cofs.2019.04.001

[B27] Payne P. Law C. Mudd E. (1980). Control by homoeologous group 1 chromosomes of the high molecular-weight subunits of glutenin, a major protein of wheat endosperm. Theor. Appl. Genet. 58, 113–120. 10.1007/BF00263101 24301341

[B28] Payne P. Nightingale M. Krattiger A. Holt L. (1987). The relationship between HMW glutenin subunit composition and the bread-making quality of British-grown wheat varieties. J. Sci. Food Agric. 40, 51–65. 10.1002/jsfa.2740400108

[B29] Peng Z. Gao Y. Chen P. Lv C. Zhao G. (2022). Recent advances in the study of wheat protein and other food components affecting the gluten network and the properties of noodles. Foods 11, 3824. 10.3390/foods11233824 36496632 PMC9738829

[B30] Rasheed A. Jin H. Xiao Y. Zhang Y. Hao Y. Zhang Y. (2019). Allelic effects and variations for key bread-making quality genes in bread wheat using high-throughput molecular markers. J. Cereal Sci. 85, 305–309. 10.1016/J.JCS.2018.12.004

[B31] Santos F. Pena S. J. Epplen J. (1993). Genetic and population study of a Y-linked tetranucleotide repeat DNA polymorphism with a simple non-isotopic technique. Hum. Genet. 90, 655–656. 10.1007/bf00202486 8444472

[B32] Sharp P. Kreis M. Shewry P. Gale M. (1988). Location of *β*-amylase sequences in wheat and its relatives. Theor. Appl. Genet. 75, 286–290. 10.1007/BF00303966

[B33] Sherman J. Varella A. Lanning S. Martin J. Heo H. Nash D. (2018). Effect of a gene for high dough strength on whole wheat baking parameters of hard white spring wheat. Cereal Chem. 95, 411–417. 10.1002/cche.10042

[B34] Shewry P. Halford N. Tatham A. Popineau Y. Lafiandra D. Belton P. (2003). The high molecular weight subunits of wheat glutenin and their role in determining wheat processing properties. Adv. Nutr. 45, 219–302. 10.1016/s1043-4526(03)45006-7 12402682

[B35] Shwry P. Tatham A. Barro F. Barcelo P. Lazzeri P. (1995). Biotechnology of breadmaking: unraveling and manipulating the multi-protein gluten complex. Nat. Biotechnol. 13, 1185–1190. 10.1038/nbt1195-1185 9636290

[B36] Sönmez M. Güleç T. Demir B. Bayraç C. Çakmak M. Aydin N. (2023). Molecular screening of the landraces from Turkey and modern bread wheat (*Triticum aestivum* L) cultivars for HMW-GS, *wbm, waxy* genes and *Lr34* gene. Genet. Resour. Crop Evol. 70, 775–788. 10.1007/s10722-022-01460-0

[B37] Wang B. Meng T. Xiao B. Yu T. Yue T. Jin Y. (2023). Fighting wheat powdery mildew: from genes to fields. Theor. Appl. Genet. 136, 196. 10.1007/s00122-023-04445-4 37606731

[B38] Wang D. Zhang K. Dong L. Dong Z. Li Y. Hussain A. (2018). Molecular genetic and genomic analysis of wheat milling and end-use traits in China: progress and perspectives. Crop J. 6, 68–81. 10.1016/j.cj.2017.10.001

[B39] Wang L. Li G. Peña R. Xia X. He Z. (2010). Development of STS markers and establishment of multiplex PCR for *Glu-A3* alleles in common wheat (*Triticum aestivum L.*). J. Cereal Sci. 51, 305–312. 10.1016/j.jcs.2010.01.005

[B40] Wang L. Zhao X. He Z. Ma W. Appels R. Peña R. (2009). Characterization of low-molecular-weight glutenin subunit *Glu-B3* genes and development of STS markers in common wheat (*Triticum aestivum* L.). Theor. Appl. Genet. 118, 525–539. 10.1007/s00122-008-0918-9 18989655

[B42] Weegels P. vande Pijpekamp A. Graveland A. Hamer R. Schofield J. (1996). Depolymerisation and re-polymerisation of wheat glutenin during dough processing.1. Relationships between glutenin macropolymer content and quality parameters. J. Cereal Sci. 23, 103–111. 10.1006/jcrs.1996.0082

[B43] Xin Q. Yin Y. Liu X. Li L. Zhao Q. Jiang H. (2019). New wheat variety‘Yannong 999’: characteristics and breeding strategy. Chin. Agric. Univ. 35, 6–10. 10.11924/j.issn.1000-6850.casb18020078

[B44] Xu T. Zhang X. Dong Y. (2006). Expression analysis of HMW-GS *1Bx14* and *1By15* in wheat varieties and transgenic research of *1By15* gene. Agr. Sci. China. 5, 725–735. 10.1016/S1671-2927(06)60117-X

[B45] Yu Z. Peng Y. Islam M. She M. Lu M. Lafiandra D. (2019). Molecular characterization and phylogenetic analysis of active y-type high molecular weight glutenin subunit genes at *Glu-A1* locus in wheat. J. Cereal Sci. 86, 9–14. 10.1016/j.jcs.2019.01.003

[B46] Zhang Y. Tang J. Yan J. Zhang Y. Zhang Y. Xia X. (2009). The gluten protein and interactions between components determine mixograph properties in an F_6_ recombinant inbred line population in bread wheat. J. Cereal Sci. 50, 219–226. 10.1016/j.jcs.2009.05.005

[B47] Zhao X. Xia X. He Z. Gale K. Lei Z. Appels R. (2006). Characterization of three low-molecular-weight *Glu-D3* subunit genes in common wheat. Theor. Appl. Genet. 113, 1247–1259. 10.1007/s00122-006-0379-y 16941095

